# Supplementary material for: Finite-momentum Cooper pairing in proximitized altermagnets
Source: Nat Commun. 2024 Feb 27;15:1801. doi: 10.1038/s41467-024-45951-3 (PMC10899178; doi:10.1038/s41467-024-45951-3)
Supplement: Supplementary file 1 — Supplementary Information [file 41467_2024_45951_MOESM1_ESM.pdf]

Supplementary Information for "Finite-momentum Cooper pairing in proximitized altermagnets"

Song-Bo Zhang,[1,2,3,*] Lun-Hui Hu,[4,5,6,†] and Titus Neupert[3]

[1] Hefei National Laboratory, Hefei, 230088, China
[2] International Center for Quantum Design of Functional Materials (ICQD), University of Science and Technology of China, Hefei, Anhui 230026, China
[3] Department of Physics, University of Zürich, Winterthurerstrasse 190, 8057, Zürich, Switzerland
[4] Department of Applied Physics, Aalto University School of Science, FI-00076 Aalto, Finland
[5] Center for Correlated Matter and School of Physics, Zhejiang University, Hangzhou 310058, China
[6] Department of Physics and Astronomy, The University of Tennessee, Knoxville, Tennessee 37996, USA

(Dated: January 20, 2024)

In this Supplementary Information, we provide the details of the derivations of the Cooper-pair propagator (Sec. I), order parameter (Sec. II), Josephson supercurrents (Sec. III), Fraunhofer patterns (Sec. IV), and correction from side edge reflection (Sec. V).

I. DERIVATION OF COOPER-PAIR PROPAGATOR

A. General formalism

The Cooper-pair propagator describes the simultaneous propagation of two electrons with opposite spin initially from a position $\mathbf{r}_1$ at time t_1 to another position $\mathbf{r}_2$ at time t_2. It can also be understood as the retarded correlation between the event of annihilating two electrons of opposite spins at 1 and another event of creating two electrons at 2. Accordingly, the Cooper-pair propagator is given by [1]

$$D(1,2) = -i\langle|T\Phi(1)\Phi^\dagger(2)|\rangle, \tag{1}$$

where $|\rangle$ is the ground state, T is time ordering, $\Phi(1)$ is the operator for an electron pair in the Heisenberg picture

$$\Phi(1) = e^{(\hat{H}_0+\hat{H}_I-\mu\hat{N})t}\Phi(\mathbf{r}_1)e^{-(\hat{H}_0+\hat{H}_I-\mu\hat{N})t}, \tag{2}$$

with $1 \equiv (\mathbf{r}_1, t_1)$, $2 \equiv (\mathbf{r}_2, t_2)$, $\Phi(\mathbf{r}) = \psi_\downarrow(\mathbf{r})\psi_\uparrow(\mathbf{r})$ and $\hat{H}_I = -\kappa\int\Phi^\dagger(\mathbf{r})\Phi(\mathbf{r})d^2\mathbf{r}$ being the pairing interaction. We assume weak electron interaction and employ Wick's theorem. Note that in the altermagnet without intrinsic pairing interaction ($\kappa = 0$), the Wick's theorem is applicable. The pair propagator can be calculated as

$$\begin{aligned} D(1,2) &= -i\langle|T\psi_\downarrow(1)\psi_\uparrow(1)[\psi_\downarrow(2)\psi_\uparrow(2)]^\dagger|\rangle = -i\langle|T\psi_\downarrow(1)\psi_\uparrow(1)\psi_\uparrow^\dagger(2)\psi_\downarrow^\dagger(2)|\rangle \\ &\approx -i[\langle|T\psi_\downarrow(1)\psi_\downarrow^\dagger(2)|\rangle\langle|T\psi_\uparrow(1)\psi_\uparrow^\dagger(2)|\rangle - \langle|T\psi_\downarrow(1)\psi_\uparrow^\dagger(2)|\rangle\langle|T\psi_\uparrow(1)\psi_\downarrow^\dagger(2)|\rangle] \\ &= iG_{0\uparrow\uparrow}(1,2)G_{0\downarrow\downarrow}(1,2) - iG_{0\uparrow\downarrow}(1,2)G_{0\downarrow\uparrow}(1,2), \end{aligned} \tag{3}$$

where $G_0(1,2) \equiv -i\langle|T\Psi(1)\Psi^\dagger(2)|\rangle$ with $\Psi^\dagger = (\psi_\uparrow^\dagger, \psi_\downarrow^\dagger)$ is the non-interacting single-particle Green's function. It is a two-by-two matrix in the spin basis. The vortex correction is ignored for simplicity. Thus, we approximate the Cooper-pair propagator by a convolution of two electron Green's functions. To work in the full spin basis, we rewrite

$$D(1,2) \approx \frac{i}{2}\mathrm{Tr}\left[\begin{pmatrix} G_{0\uparrow\uparrow}(1,2) & G_{0\uparrow\downarrow}(1,2) \\ G_{0\downarrow\uparrow}(1,2) & G_{0\downarrow\downarrow}(1,2)\end{pmatrix}\begin{pmatrix} 0 & -i \\ i & 0\end{pmatrix}\begin{pmatrix} G_{0\uparrow\uparrow}(1,2) & G_{0\downarrow\uparrow}(1,2) \\ G_{0\uparrow\downarrow}(1,2) & G_{0\downarrow\downarrow}(1,2)\end{pmatrix}\begin{pmatrix} 0 & -i \\ i & 0\end{pmatrix}\right] = \frac{i}{2}\mathrm{Tr}\left[G_0(1,2)s_yG_0^T(1,2)s_y\right]. \tag{4}$$

If spin is a good quantum number, then the electron Green's function is diagonal, $G_0(1,2) = G_0^T(1,2)$. Otherwise, (e.g., in the presence of in-plane Zeeman field or spin-orbit interaction), the off-diagonal terms appear, indicating that the electrons could vary their spin during the propagation.

Our aim is to evaluate the pair propagator at frequency ω which is the Fourier transform of $D(1,2)$

$$D(\mathbf{r}_1;\mathbf{r}_2;\omega) = \int_{-\infty}^{\infty} dt e^{i\omega t}D(1,2), \quad t = t_2 - t_1. \tag{5}$$

$D(\mathbf{r}_1;\mathbf{r}_2;i\nu_m) =$ 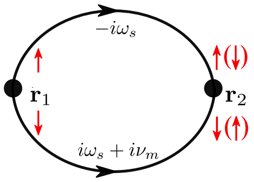

Fig. 1. Diagram for a propagator for an electron pair. The vertex correction due to the scattering of the electrons is not considered for simplicity. The red arrows indicate opposite spins carried by the electron pair. Note that in the presence of Zeeman or spin-orbit interactions, the electrons could vary their spin during the propagation.

It is usually easiest to calculate the retarded correlation function (here the pair propagator) in the Matsubara formalism. Using the Feymann diagram techniques, the Matsubara pair propagator $D(\mathbf{r}_1;\mathbf{r}_2;i\nu_m)$ can be evaluated as Fig. 1. (Note that $D(\mathbf{r}_1;\mathbf{r}_2,\omega)$ can be obtained from $D(\mathbf{r}_1;\mathbf{r}_2;i\nu_m)$ by performing analytic continuation: $i\nu_m \to \omega + i0^+$ [2].) Thus, it can be written as

$$D(\mathbf{r}_1;\mathbf{r}_2;i\nu_m) = \frac{1}{2\beta}\sum_{\omega_s} \mathrm{Tr}[\mathcal{G}_0(\mathbf{r}_1,\mathbf{r}_2,-i\omega_s)s_y\mathcal{G}_0^T(\mathbf{r}_1,\mathbf{r}_2,i\omega_s+i\nu_m)s_y], \tag{6}$$

where $\mathcal{G}_0(\mathbf{r}_1,\mathbf{r}_2,i\omega_s)$ is the Matsubara Green's function, $\omega_s = (2s+1)\pi k_B T$ and $\nu_m = 2\pi m k_B T$ (with s and m being integers) are Matsubara frequencies for fermions and bosons (here electron pairs). Assuming translation symmetry in the system, we further transform the electron Green's functions into momentum space. As a result, we have

$$D(\mathbf{r},i\nu_m) = \frac{1}{2\beta}\sum_{\omega_s}\int\frac{d^2\mathbf{k}}{(2\pi)^2}e^{i\mathbf{k}\cdot\mathbf{r}}\int\frac{d^2\mathbf{k}'}{(2\pi)^2}e^{i\mathbf{k}'\cdot\mathbf{r}}\mathrm{Tr}[\mathcal{G}_0(\mathbf{k},-i\omega_s)s_y\mathcal{G}_0^T(\mathbf{k}',i\omega_s+i\nu_m)s_y], \tag{7}$$

where $\mathbf{r} = \mathbf{r}_2 - \mathbf{r}_1$. The Matsubara Green's functions can be related to the spectral functions $A_0(\mathbf{k},\epsilon)$ by [2]

$$\mathcal{G}_0(\mathbf{k},i\omega_s) = \int_{-\infty}^{\infty}\frac{d\epsilon}{2\pi}\frac{A_0(\mathbf{k},\epsilon)}{i\omega_s-\epsilon}. \tag{8}$$

Therefore, we have

$$D(\mathbf{r},i\nu_m) = \frac{1}{2\beta}\int_{-\infty}^{\infty}\frac{d\epsilon}{2\pi}\frac{d\epsilon'}{2\pi}\mathcal{T}(\mathbf{r},\epsilon,\epsilon')\sum_{\omega_s}\frac{1}{-i\omega_s-\epsilon}\frac{1}{i\omega_s+i\nu_m-\epsilon'}, \tag{9}$$

where we have defined the Fourier transform of the spectral function as

$$g_0(\mathbf{r},\epsilon) = \int\frac{d^2\mathbf{k}}{(2\pi)^2}e^{i\mathbf{k}\cdot\mathbf{r}}A_0(\mathbf{k},\epsilon), \tag{10}$$

and

$$\mathcal{T}(\mathbf{r},\epsilon,\epsilon') = \mathrm{Tr}[g_0(\mathbf{r},\epsilon)s_y g_0^T(\mathbf{r},\epsilon')s_y]. \tag{11}$$

The frequency summation over ω_s gives

$$\frac{1}{\beta}\sum_{\omega_s}\frac{1}{-i\omega_s-\epsilon}\frac{1}{i\omega_s+i\nu_m-\epsilon'} = -\frac{n_F(-\epsilon)-n_F(\epsilon')}{i\nu_m-\epsilon-\epsilon'}, \tag{12}$$

where $n_F(E) = 1/[1+\exp(\beta E)]$ is the Fermi distribution function. Thus, $D(1,2)$ becomes

$$D(\mathbf{r},i\nu_m) = \frac{1}{2}\int_{-\infty}^{\infty}\frac{d\epsilon}{2\pi}\frac{d\epsilon'}{2\pi}\mathcal{T}(\mathbf{r},\epsilon,\epsilon')\frac{n_F(\epsilon')-n_F(-\epsilon)}{i\nu_m-\epsilon-\epsilon'}. \tag{13}$$

In the low-temperature ($T=0$) limit, we have $n_F(E) = 1$ if $E < 0$, and $n_F(E) = 0$ if $E > 0$. Hence,

$$D(\mathbf{r},i\nu_m) = \frac{1}{2}\int_0^{\infty}\frac{d\epsilon}{2\pi}\frac{d\epsilon'}{2\pi}\frac{\mathcal{T}(\mathbf{r},\epsilon,\epsilon')}{-i\nu_m+\epsilon+\epsilon'} + \frac{1}{2}\int_0^{\infty}\frac{d\epsilon}{2\pi}\frac{d\epsilon'}{2\pi}\frac{\mathcal{T}(\mathbf{r},-\epsilon,-\epsilon')}{i\nu_m+\epsilon+\epsilon'}. \tag{14}$$

Performing analytic continuation $i\nu_m \to \omega + i0^+$, we obtain

$$D(\mathbf{r},\omega) = \frac{1}{2}\int_0^\infty \frac{d\epsilon}{2\pi}\frac{d\epsilon'}{2\pi}\frac{\mathcal{T}(\mathbf{r},\epsilon,\epsilon')}{-\omega - i0^+ + \epsilon + \epsilon'} + \frac{1}{2}\int_0^\infty \frac{d\epsilon}{2\pi}\frac{d\epsilon'}{2\pi}\frac{\mathcal{T}(\mathbf{r},-\epsilon,-\epsilon')}{\omega + i0^+ + \epsilon + \epsilon'}. \tag{15}$$

Finally, we consider the static limit $\omega = 0$ and arrive at

$$D(\mathbf{r}) = D(\mathbf{r},0) = \frac{1}{2}\int_0^\infty \frac{d\epsilon}{2\pi}\frac{d\epsilon'}{2\pi}\frac{\mathcal{T}(\mathbf{r},\epsilon,\epsilon') + \mathcal{T}(\mathbf{r},-\epsilon,-\epsilon')}{\epsilon + \epsilon'}. \tag{16}$$

B. Application to altermagnet

To derive the Cooper-pair propagator in the altermagnet, we first calculate the spectral function in real space. Defining the projection matrices as

$$P_\eta = \frac{1 + \eta s_z}{2}, \tag{17}$$

the non-interacting retarded Green's function can be written as

$$G_0^{\rm ret}(\mathbf{k},\epsilon) = \sum_\eta \frac{P_\eta}{\epsilon - \varepsilon_{\mathbf{k},\eta} + i\delta}. \tag{18}$$

Thus, the spectral function in momentum space reads

$$A_0(\mathbf{k},\epsilon) \equiv -2\mathrm{Im}[G_0^{\rm ret}(\mathbf{k},\epsilon)] = 2\pi\sum_\eta \delta(\epsilon - \varepsilon_{\mathbf{k},\eta})P_\eta. \tag{19}$$

Fourier transforming Eq. (19), we find the spectral function in real space as

$$\begin{aligned} g_0(\mathbf{r},\epsilon) &= 2\pi\sum_\eta\int\frac{d^2\mathbf{k}}{(2\pi)^2}e^{i\mathbf{k}\cdot\mathbf{r}}\delta(\epsilon - \varepsilon_{\mathbf{k},\eta})P_\eta \\ &= \frac{1}{2\pi}\sum_\eta\int_0^\infty dkk\int_0^{2\pi}d\phi e^{ikr\cos(\phi-\theta)}\frac{\delta(k-k_\eta)}{|\partial\varepsilon_{\mathbf{k},\eta}/\partial_k|_{k=k_\eta}}P_\eta \\ &= \frac{1}{2\pi}\sum_\eta k_\eta\int_0^{2\pi}d\phi e^{ik_\eta r\cos(\phi-\theta)}\frac{P_\eta}{k_\eta[1+\eta J\sin(2\phi)/2]|} \\ &= \frac{1}{2\pi}\sum_\eta\int_0^{2\pi}d\phi' e^{ik_\eta r\cos\phi'}\frac{P_\eta}{1+\eta J\sin(2\phi'+2\theta)/2}, \end{aligned} \tag{20}$$

where we have changed the integral to polar coordinates in the second line, redefined the angle variable $\phi' \equiv \phi - \theta$ in the last line, and the Fermi wave numbers are given by

$$k_\eta = \sqrt{\frac{\epsilon + \mu}{1 + \eta J\sin(2\phi)/2}}. \tag{21}$$

In this work, we focus on the situation with a large chemical potential such that $\mu \gg |\epsilon|$ and $k_\eta r \gg 1$. In this case, the factor $e^{ik_\eta r\cos\phi'}$ in Eq. (20) is a rapidly oscillating function of ϕ'. We apply the saddle-point approximation to the integration over ϕ' by expanding $k_\eta r\cos\phi'$ around its extremal points near which the integration is important [3]. As a result, we obtain

$$g_0(\mathbf{r},\epsilon) \;\approx\; \frac{1}{(2\pi r)^{1/2}\mu^{1/4}}\sum_\eta\frac{P_\eta}{[1+\eta J\sin(2\theta)/2]^{3/4}}(e^{i\bar{k}_\eta r - i\pi/4} + e^{-i\bar{k}_\eta r + i\pi/4}), \tag{22}$$

where $\bar{k}_\eta$ can be approximated as

$$\bar{k}_\eta = \sqrt{\frac{\epsilon+\mu}{1+\eta J\sin(2\theta)/2}} \approx \sqrt{\frac{2\mu}{2+\eta J\sin(2\theta)}}\Big(1 + \frac{\epsilon}{2\mu}\Big). \tag{23}$$

The signs in the factor $e^{\mp i\pi/4}$ for the two extremal points of $k_\eta r\cos\phi'$ at $\phi'=0$ and π are negative and positive, respectively, because $(\partial^2\cos\phi'/\partial\phi'^2)|_{\phi'=0}<0$ and $(\partial^2\cos\phi'/\partial\phi'^2)|_{\theta=\pi}>0$.

Plugging Eq. (22) into Eq. (11), we have

$$\mathcal{T}(\mathbf{r},\epsilon,\epsilon')=\frac{1}{2\pi r\mu^{1/2}}\sum_{\eta,\eta'}\frac{(e^{i\bar{k}_\eta r-i\pi/4}+e^{-i\bar{k}_\eta r+i\pi/4})(e^{i\bar{k}'_{\eta'}r-i\pi/4}+e^{-i\bar{k}'_{\eta'}r+i\pi/4})}{\{[1+\eta J\sin(2\theta)/2][1+\eta' J\sin(2\theta)/2]\}^{3/4}}\mathrm{Tr}[P_\eta s_y P_{\eta'} s_y]. \tag{24}$$

For the trace, we find

$$\mathrm{Tr}[P_\eta s_y P_{\eta'} s_y]=\delta_{\eta',-\eta}. \tag{25}$$

Only the terms with different spin indexes $\eta\neq\eta'$ contribute to the propagator. This is expected as an s-wave Cooper pair consists of two electrons of opposite spins. Therefore, we arrive at

$$\mathcal{T}(\mathbf{r},\epsilon,\epsilon')=\frac{2^{3/2}}{\pi r\mu^{1/2}[4-J^2\sin^2(2\theta)]^{3/4}}\sum_\eta\{\cos[(\bar{k}_\eta-\bar{k}'_{-\eta})r]+\sin[(\bar{k}_\eta+\bar{k}'_{-\eta})r]\}, \tag{26}$$

and hence

$$\begin{aligned}&\mathcal{T}(\mathbf{r},\epsilon,\epsilon')+\mathcal{T}(\mathbf{r},-\epsilon,-\epsilon')\\ =&\frac{2^{5/2}}{\pi r\mu^{1/2}[4-J^2\sin^2(2\theta)]^{3/4}}\Big\{\cos[\sqrt{2\mu}(J_+-J_-)r]\Big[\cos\Big(\frac{J_+\epsilon-J_-\epsilon'}{\sqrt{2\mu}}r\Big)+\cos\Big(\frac{J_-\epsilon-J_+\epsilon'}{\sqrt{2\mu}}r\Big)\Big]\\ &+\sin[\sqrt{2\mu}r(J_++J_-)]\Big[\cos\Big(\frac{J_+\epsilon+J_-\epsilon'}{\sqrt{2\mu}}r\Big)+\cos\Big(\frac{J_-\epsilon+J_+\epsilon'}{\sqrt{2\mu}}r\Big)\Big]\Big\},\end{aligned} \tag{27}$$

where

$$J_\pm=\frac{1}{\sqrt{2\pm J\sin(2\theta)}}. \tag{28}$$

Plugging Eq. (27) into Eq. (16), we have finally

$$D(\mathbf{r})=\frac{(J_+J_-)^{3/2}}{\pi^2r^2(J_++J_-)}(e^{iqr}+e^{-iqr}), \tag{29}$$

where we have considered $a=J_+/J_->0$ (i.e., $|J|<2$), defined $q=\sqrt{2\mu}(J_+-J_-)$, and used

$$\int_0^\infty dxdy\frac{\cos(ax-y)}{x+y}=\frac{\pi}{1+a},\quad \int_0^\infty dxdy\frac{\cos(ax+y)}{x+y}=0. \tag{30}$$

In the derivation, we notice that (J_+-J_-) pairing is allowed while (J_++J_-) not. This result is related to the integral over the excitation energies ϵ and ϵ'. Physically speaking, this indicates that two electrons of opposite spins can pair up and propagate if they have the same sign of velocity in the direction of propagation. Whereas if they have opposite signs of velocity, they are not likely to pair and propagate.

II. DERIVATION OF THE ORDER PARAMETER

We first perform numerical calculations of the local order parameter $\langle|\Psi(\mathbf{r}')|\rangle$ by directly integrating Eq. (4) in the main text. The numerical results are displayed in Fig. 2. Interestingly, we find that when the interface (junction) width and chemical potential energy in the altermagnet are large, i.e., $W\gg y'\gg 1/(J\sqrt{\mu})$, the order parameter $\langle|\Psi(\mathbf{r}')|\rangle$ (not close to the edges at $x'=\pm W/2$) becomes independent of W and position x along the interface. This result holds for interfaces in any orientation. Thus, it suffices to calculate the order parameter at $x'=0$ which reads

$$\langle|\Psi(0,y')|\rangle=\frac{2\lambda}{\pi^2}\int_{-W/2}^{W/2}dx'_1\frac{(J'_+J'_-)^{3/2}}{r'^2(J'_++J'_-)}\cos[\sqrt{2\mu}r'(J'_--J'_+)], \tag{31}$$

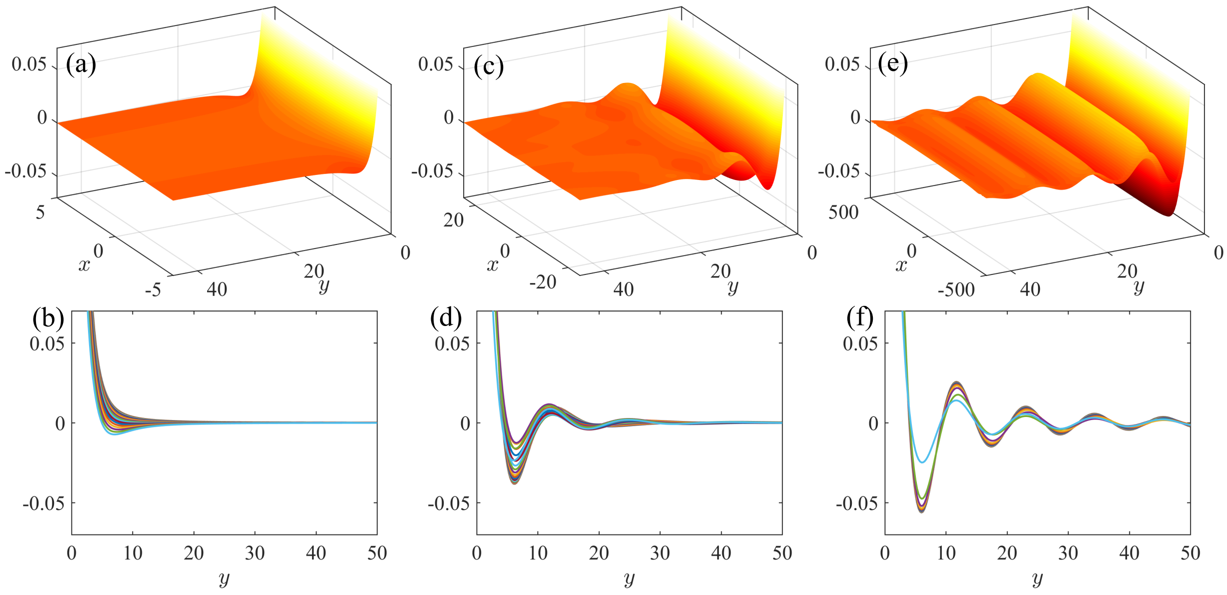

Fig. 2. Order parameter $\langle|\Psi(\mathbf{r})|\rangle$ (in units of λ/π^2) induced from an s-wave superconductor by proximity effect for widths $W = 10$ (a,b), 50 (c,d) and 1000 (e,f), respectively. The interface is in x direction and y is the distance from the interface. We see that the order parameter becomes independent of x, and damped oscillations as a function y appear for $W \gg y$. Other parameters are $\varphi = 0$, $J = 0.8$ and $\mu = 0.5$.

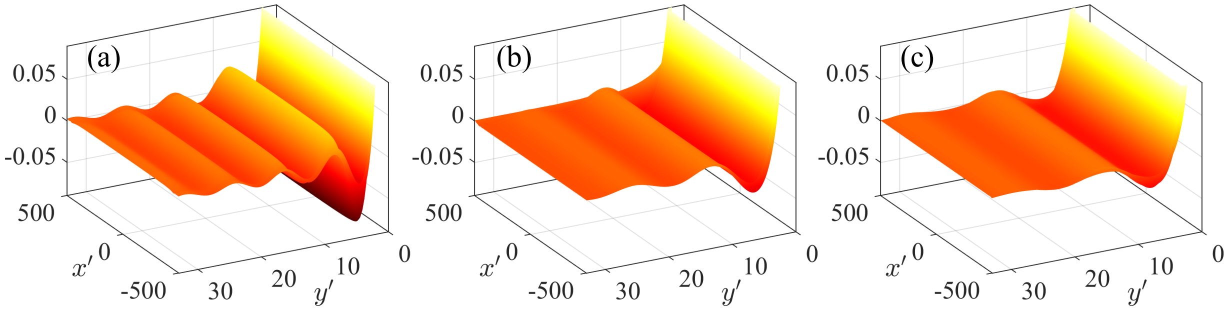

Fig. 3. Local order parameter $\langle|\Psi(\mathbf{r}')|\rangle$ (in units of λ/π^2) for different junction orientations $\varphi = 0$ (a), 0.1π (b) and 0.25π (c), respectively. The order parameter is nearly constant in x' but oscillates with y'.

where

$$J'_{\pm} = \frac{1}{\sqrt{2 \pm J\sin(2\theta' + 2\varphi)}}. \tag{32}$$

Converting the integral over x' to an integral over the angle θ' (i.e., $dx' = -\csc^2\theta' d\theta'$), we have

$$\langle|\Psi(0,y')|\rangle = \frac{2\lambda}{\pi^2}\frac{1}{y'}\int_{\alpha}^{\pi-\alpha} d\theta' \frac{(J'_{+}J'_{-})^{3/2}}{J'_{+}+J'_{-}}\cos(\sqrt{\mu}\mathcal{F}_{\theta'}y'), \tag{33}$$

where $\alpha = \arctan(2y'/W)$ and $\mathcal{F}_{\theta'} = \sqrt{2}\csc\theta'(J'_{-} - J'_{+})$. For large $y' \gg 1/(J\sqrt{\mu})$, the function $\cos(\sqrt{\mu}y'\mathcal{F}_{\theta'})$ oscillates rapidly as θ' varies. Thus, we apply the saddle point approximation to the integral over θ' and obtain

$$\langle|\Psi(0,y')|\rangle \approx \lambda\left(\frac{2}{\pi y'}\right)^{3/2}\left\{\frac{(J'_{+}J'_{-})^{3/2}}{J'_{+}+J'_{-}}\frac{\cos(\sqrt{\mu}\mathcal{F}_{\theta'}y' + \pi/4)}{|\sqrt{\mu}\partial^2\mathcal{F}_{\theta'}/\partial\theta'^2|^{1/2}}\right\}\Big|_{\theta'=\theta_m}, \tag{34}$$

where θ_m is the minimum point of $\mathcal{F}_{\theta'}$. We see clearly that the for given μ, the oscillations in y' are more rapid when the interface is along x-direction. This is in good agreement with the numerical calculations shown in Fig. 3.

III. DERIVATION OF JOSEPHSON SUPERCURRENT

In this section, we derive the formula for Josephson supercurrent. The pairing interaction of the planar junction can be written as

$$H_p = -\int d^2\mathbf{r}'[\Delta(\mathbf{r}')\Psi^\dagger(\mathbf{r}') + \Delta^*(\mathbf{r}')\Psi(\mathbf{r}')], \tag{35}$$

where $\Psi(\mathbf{r}') \equiv \psi_\uparrow(\mathbf{r}')\psi_\downarrow(\mathbf{r}')$ and $\Delta(\mathbf{r}')$ is the pairing potential. We assume

$$\Delta(\mathbf{r}') = \lambda_1(x')\delta(y') + \lambda_2(x')\delta(y' - L), \tag{36}$$

with $|x'| < W/2$ and that the magnitude of $\lambda_j > 0$ (with $j \in \{1, 2\}$) is constant along the superconducting lead, $\lambda_j(x') = \lambda_j e^{i\phi_j}$.

We are interested in supercurrents which can be evaluated by the derivative of free energy with respect to the pairing phase difference $\delta_\phi = \phi_1 - \phi_2$ across the junction. The free energy contributed by the pairing interaction, $F_p = \langle|H_p|\rangle$, is given by

$$F_p = -\int dx'[\lambda_1(x')\langle|\Psi^\dagger(x', 0)|\rangle + \lambda_2(x')\langle|\Psi^\dagger(x', L)|\rangle + \lambda_1^*(x')\langle|\Psi(x', 0)|\rangle + \lambda_2^*(x')\langle|\Psi(x', L)|\rangle]. \tag{37}$$

Here, $\langle|\Psi(\mathbf{r}')|\rangle$ is the order parameter, induced from the two superconducting leads by proximity effect. We may write it as two parts $\langle|\Psi(\mathbf{r}')|\rangle = \langle|\Psi(\mathbf{r}')|\rangle_1 + \langle|\Psi(\mathbf{r}')|\rangle_2$ with

$$\begin{aligned}\langle|\Psi(\mathbf{r}')|\rangle_1 &= \int dx_1'\lambda_1(x_1')D(\mathbf{r}'; x_1', 0),\\ \langle|\Psi(\mathbf{r}')|\rangle_2 &= \int dx_1'\lambda_2(x_1')D(\mathbf{r}'; x_1', W).\end{aligned} \tag{38}$$

Plugging Eq. (38) into Eq. (37), the $\delta\phi$-dependent part of the free energy is found as

$$F_{\delta_\phi} = -2\lambda_1\lambda_2\int dx'dx_1'[e^{-i\delta_\phi}D(x', 0; x_1', L) + e^{i\delta_\phi}D(x', L; x_1', 0)]. \tag{39}$$

The supercurrent is then obtained as

$$I(\delta_\phi) = \frac{e}{\hbar}\frac{\partial F_{\delta_\phi}}{\partial\delta_\phi} = -i\frac{2e}{\hbar}\lambda_1\lambda_2\int_{-W/2}^{W/2} dx'dx_1'[-e^{-i\delta_\phi}D(x', 0; x_1', L) + e^{i\delta_\phi}D(x', L; x_1', 0)] \tag{40}$$

Exchanging dummy variables, we can replace $D(x', 0; x_1', L)$ by $D(x', L; x_1', 0)$. Using the fact that the propagator is real valued, the supercurrent can be finally written as

$$I(\delta_\phi) = I_c\sin(\delta_\phi), \tag{41}$$

where the critical supercurrent reads

$$I_c = \frac{4e}{\hbar}\lambda_1\lambda_2\int_{-W/2}^{W/2} dx'dx_1'D(x', L; x_1', 0). \tag{42}$$

For $W \gg \{L, 1/J\sqrt{\mu}\}$, $\int dx_1'D(x', L; x_1', 0)$ can be evaluated in a similar way as the order parameter $\langle|\Psi(\mathbf{r}')|\rangle$. It turns out to be constant in x'. Thus, I_c can be simplified to

$$I_c = \frac{4e}{\hbar}\lambda_1\lambda_2 W\Big(\frac{2J_+'J_-'}{\pi L}\Big)^{3/2}\frac{\cos(\sqrt{\mu}\mathcal{F}_{\theta'}L + \pi/4)}{(J_+' + J_-')|\sqrt{\mu}\partial^2\mathcal{F}_{\theta'}/\partial\theta'^2|^{1/2}}\Big|_{\theta'=\theta_m}. \tag{43}$$

IV. CALCULATIONS OF THE FRAUNHOFER PATTERN

In this section, we consider a uniform perpendicular magnetic field B applied to the altermagnetic metal region of the planar Josephson junction and calculate the resulting Fraunhofer pattern. Under the magnetic field, the coupling

magnitudes λ (with $j \in \{1,2\}$) are constant but the phases ϕ_j vary along the interface. Adopting the Landau gauge $\mathbf{A} = -B(y' - L/2, 0, 0)$, we have

$$\lambda_j(x') = \lambda_j e^{i\phi_j(x')} \tag{44}$$

in Eq. (36), where

$$\begin{aligned} \phi_1(x') &= \phi_1(0) + \frac{e}{\hbar}\int_0^{x'} A_x(x'', 0)dx'' = \phi_1(0) + \alpha x' B, \\ \phi_2(x') &= \phi_2(0) + \frac{e}{\hbar}\int_0^{x'} A_x(x'', L)dx'' = \phi_2(0) - \alpha x' B, \end{aligned} \tag{45}$$

with $\alpha \equiv 2\pi L/(2\Phi_0)$, $\Phi_0 = h/e$ is the electronic flux quantum, $\phi_1(0)$ and $\phi_2(0)$ are the pairing phases in the first and second superconducting leads in the absence of the applied magnetic field, respectively.

Following the same procedure as that in the previous section, we derive the δ_ϕ-dependent part of the free energy as

$$F_{\delta_\phi} = -2\int_{-W/2}^{W/2} dx' dx'_1 \mathrm{Re}[\lambda_2^*(x')\lambda_1(x'_1)D(x', L; x'_1, 0) + c.c.]. \tag{46}$$

Here, $c.c.$ means complex conjugation. Hence, the Josephson current is obtained as

$$I(\delta_\phi) = \frac{e}{\hbar}\frac{\partial F_{\delta_\phi}}{\partial \delta_\phi} = \frac{4e}{\hbar}\lambda_1\lambda_2 \mathrm{Im}\Big[e^{i\delta_\phi}\int_{-W/2}^{W/2} dx' dx'_1 e^{i\alpha B(x'+x'_1)} D(x', L; x'_1, 0)\Big]. \tag{47}$$

The Fraunhofer interference pattern shows the maximal value of $I(\delta_\phi)$ (with respect to δ_ϕ) as a function of the applied magnetic flux (magnetic field B). Using Eq. (47), the maximum supercurrent can be found as

$$I_m(B) = \frac{4e}{\hbar}\max[I(\delta_\phi)] = \frac{4e}{\hbar}\lambda_1\lambda_2\Big|\int_{-W/2}^{W/2} dx' dx'_1 e^{i\alpha B(x'+x'_1)} D(x', L; x'_1, 0)\Big|. \tag{48}$$

After integration on one of the two coordinates, say x'_1, we can rewrite Eq. (48) as

$$I_m(B) = \Big|\int_{-W/2}^{W/2} dx' e^{i\alpha B x'} j_c(x', B)\Big|, \tag{49}$$

where $j_c(x', B) = \frac{4e\lambda_1\lambda_2}{\hbar}\int_{-W/2}^{W/2} dx'_1 e^{i\alpha B x'_1} D(x', L; x'_1, 0)$ defines the critical-current density per unit length along x'.

V. CONTRIBUTION OF SIDE EDGE REFLECTIONS

In this section, we consider the contribution of the reflection from the edge of the sample, at $x = \pm W/2$. In the presence of edge reflection, the Cooper-pair propagator may be modified as

$$D_{tot}(x_2, L; x_1, 0) = D(\mathbf{r}) + D_L(x_2, L; x_1, 0) + D_R(x_2, L; x_1, 0), \tag{50}$$

where D_L and D_R represent the corrections by the reflection at $x = \mp W/2$, respectively. Here, we use x_1 and x_2 to denote the coordinates along the two interfaces at $y' = 0$ and L, respectively. Suppose the mirror symmetry in the system geometry with respect to the center $x = 0$ of the system, D_L and D_R are of the same form. It is sufficient to derive D_L. For concreteness and simplicity, we consider a junction with $\varphi = \pi/4$ and assume that the electrons are specularly reflected at the edge only once and without flipping spin, as shown in Fig. 4. Similar to the case with translational invariance, we may write D_L in the from

$$\begin{aligned} &D_L(x_2, L; x_1, 0) \\ &= \int_0^\infty d\epsilon d\epsilon' \frac{\mathrm{Tr}[h(x_2, L; x_1, 0; \epsilon)s_y h^T(x_2, L; x_1, 0; \epsilon')s_y] + \mathrm{Tr}[h(x_2, L; x_1, 0; -\epsilon)s_y h^T(x_2, L; x_1, 0; -\epsilon')s_y]}{2(\epsilon + \epsilon')}, \end{aligned} \tag{51}$$

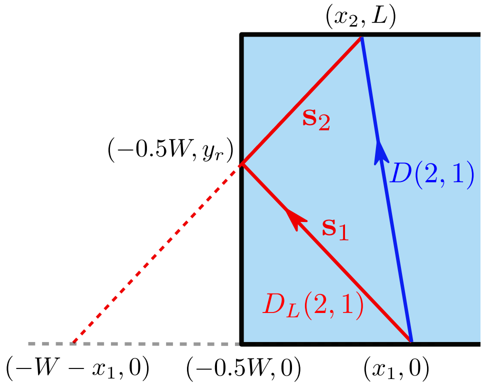

Fig. 4. The blue and red lines represent the fast trajectories in the propagators $D(2,1)$ and $D_L(2,1)$, respectively. D_L contains one specular reflection by the edge boundary. $\mathbf{s}_1$ and $\mathbf{s}_2$ denote the trajectories before and after the reflection, respectively.

where $h(x_2, L; x_1, 0; \epsilon)$ is the spectral function that corresponds to the Green's function of an electron moving from the point $(x_1, 0)$ to the point (x_2, L) via the specular reflecting point at $(-W/2, y_r)$ with $y_r = L(x_1+W/2)/(x_1+x_2+W)$. The spectral function may be calculated as

$$h(x_2, L; x_1, 0; \epsilon) = \frac{1}{2\pi}\sum_\eta \int_0^{2\pi} d\phi e^{i(k_{1\eta}s_1+k_{2\eta}s_2)\cos(\phi-\theta)} \frac{1}{2[1+\eta J\sin(2\phi)/2]} P_\eta, \tag{52}$$

where $\mathbf{s}_1 = (-x_1, y_r)$, $\mathbf{s}_2 = (x_2, L - y_r)$, θ is the direction of $\mathbf{s_1}$, and

$$k_{1\eta} = \sqrt{\frac{\epsilon+\mu}{1+\eta J\sin(2\phi)/2}}, \quad k_{2\eta} = \sqrt{\frac{\epsilon+\mu}{1+\eta J\sin(\pi-2\phi)/2}}. \tag{53}$$

In the first line of Eq. (52), the delta functions $\delta(k_{1x}+k_{2x})$ and $\delta(k_{1y}-k_{2y})$ arise due to the fact that the reflection is specular. Thus, the angle between $\mathbf{k}_1$ and $\mathbf{s}_1$ is identical to the one between $\mathbf{k}_2$ and $\mathbf{s}_2$. The other delta function $\delta(\epsilon - \varepsilon_{\mathbf{k}_1\eta_1})$ indicates the energy conservation. In the last line, we have transformed the variables in the integral to polar coordinates and integrated over the magnitude of the momentum.

Similarly, we consider the case with $k_{1\eta}s_1 + k_{2\eta}s_2 \gg 1$ and apply the saddle point approximation to the integration over θ. This results in

$$h(x_2, L; x_1, 0; \epsilon) \approx \frac{1}{(2\pi s)^{1/2}\mu^{1/4}}\sum_\eta \frac{P_\eta}{[1+\eta J\sin(2\theta)/2]^{3/4}}(e^{i\bar{k}_\eta s - i\pi/4} + e^{-i\bar{k}_\eta s + i\pi/4}), \tag{54}$$

where $s = s_1 + s_2$ and $\bar{k}_\eta$ is given by Eq. (23). Note that when the initial $(x_1, 0)$ or final (x_2, L) point approach to the boundary at $x = -W/2$, the reflection point coincides the initial or final point. In this case, we see that Eq. (54) recovers the result in Eq. (22). Plugging Eq. (54) and following the same procedures as before, we obtain

$$D_L(x_2, L; x_1, 0) = \frac{2\cos[\sqrt{2\mu}(J_+ - J_-)s]}{\pi^2 s^2 (J_+ + J_-)[4 - J^2\sin^2(2\theta)]^{3/4}}. \tag{55}$$

It is important to note that the propagator decay quadratically with the propagation distance s. Thus, for $L < W$, the correction due to the Cooper pair propagators with an edge reflection would be much smaller compared to those without an edge reflection. To confirm this, we calculate numerically the supercurrent in junctions with different sizes and compare the results with and without side edge reflections. As shown in Fig. 5, we see that for $W \lesssim L$, the corrections from side edge reflections become ignorable.

* songbozhang@ustc.edu.cn
† hu.lunhui.zju@gmail.com
[1] P. A. Lee and M. G. Payne, "Pair propagator approach to fluctuation-induced diamagnetism in superconductors-effects of impurities", Phys. Rev. B **5**, 923 (1972).
[2] G. D. Mahan, Many-Particle Physics (Plenum Press, New York, 1990).
[3] S.-B. Zhang and J. Zhou, "Quantum oscillations in acoustic phonons in weyl semimetals", Phys. Rev. B **101**, 085202 (2020).

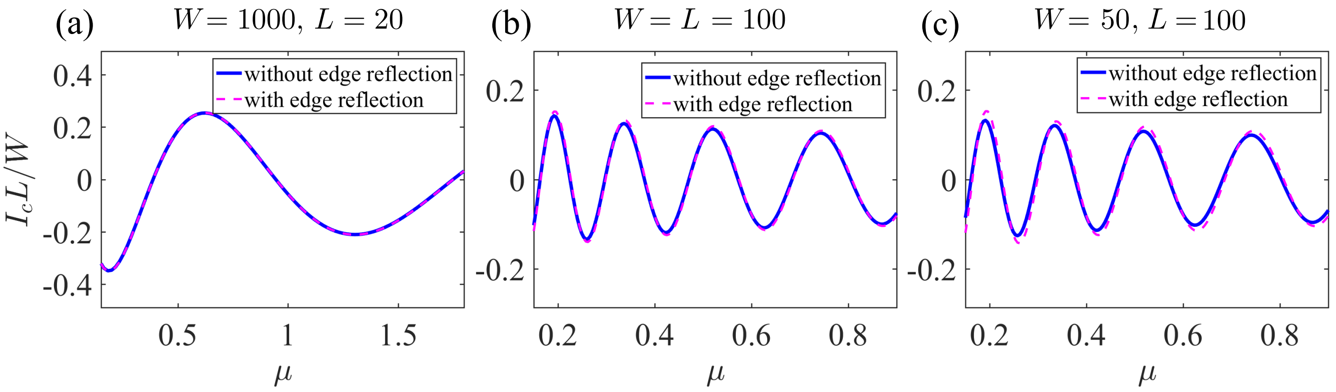

Fig. 5. Critical supercurrent density I_cL/W (in units of $4e\lambda_1\lambda_2/\pi^2\hbar$) as a function of chemical potential μ for $\varphi = \pi/4$. We consider $(W, L) = (1000, 20)$, $(W, L) = (100, 100)$ and $(W, L) = (100, 50)$ for (a), (b) and (c), respectively. The blue solid and purple dash curves present the results without and with side edge reflections. For $W \lesssim L$, the corrections from side edge reflections become ignorable.
